# Emerging Roles of Long Non-Coding RNAs in Ankylosing Spondylitis

**DOI:** 10.3389/fimmu.2022.790924

**Published:** 2022-02-10

**Authors:** Ruifu Sun, Xuesong Wang, Xiaohong Sun, Bing Zhao, Xiugong Zhang, Xiaojin Gong, Sunny Hei Wong, Matthew Tak Vai Chan, William Ka Kei Wu

**Affiliations:** ^1^Department Spinal of Qingdao Hospital Central, Qingdao Hospital Central, Qingdao, China; ^2^Department Obstetrics and Gynecology of Qingdao Hospital Central, Central Qingdao Hospital, Qingdao, China; ^3^Lee Kong Chian School of Medicine, Nanyang Technological University, Singapore, Singapore; ^4^State Key Laboratory of Digestive Disease and LKS Institute of Health Sciences, The Chinese University of Hong Kong, Hong Kong, Hong Kong SAR, China; ^5^Department of Anaesthesia and Intensive Care and Peter Hung Pain Research Institute, The Chinese University of Hong Kong, Hong Kong, Hong Kong SAR, China

**Keywords:** lncRNAs, ankylosing spondylitis, inflammatory cytokine, microRNAs, biomarkers

## Abstract

Ankylosing spondylitis (AS) is a chronic systemic autoimmune disease characterized by inflammation, bone erosion, spur formation of the spine and the sacroiliac joints. However, the etiology and molecular pathogenesis of AS remain largely unclear. Recently, a growing number of studies showed that long non-coding RNAs (lncRNAs) played critical roles in the development and progression of autoimmune and orthopedic conditions, including AS. Studies demonstrated that a myriad of lncRNAs (e.g. H19, MEG3, LOC645166) pertinent to regulation of inflammatory signals were deregulated in AS. A number of lncRNAs might also serve as new biomarkers for the diagnosis and predicting the outcomes of AS. In this review, we summarize lncRNA profiling studies on AS and the functional roles and mechanism of key lncRNAs relevant to AS pathogenesis. We also discuss their potential values as biomarkers and druggable targets for this potentially disabling condition.

## Introduction

Ankylosing spondylitis (AS) is a chronic systemic autoimmune-mediated inflammatory disease, which primarily affects the spine and the sacroiliac joints and causes pain and stiffness of these joints (1–5). Some patients with chronic AS further develop ankylosis and spinal immobility, significantly reducing their quality of life (6–8). AS mostly presents before the age of 45. Although AS has no known specific cause, individuals harbouring *HLA-B27* (one among hundreds of different forms the *HLA-B* gene) are at a much greater risk (9, 10). The molecular pathogenesis is complex and involves genetic, immunological, microbial (e.g., high abundance of *Klebsiella pneumoniae* in the gut microbiome) and hormonal (e.g., sex hormone disturbance, glucocorticoid deficiency) components (11). *HLA-B27* accounts for 20% of the total AS heritability. It encodes a major histocompatibility complex (MHC) Class I protein that is prone to misfolding in the endoplasmic reticulum (ER) and abnormal dimerization on cell surface as compared to proteins encoded by other *HLA* loci. Such misfolding and dimerization can lead to ER stress and activation of KIR3DL2 receptors expressed on immune cells [e.g., natural killer (NK) cells and T helper 17 (Th17) cells], respectively. It has also been proposed that HLA-B27 might preferentially present arthritogenic peptides to CD8^+^ T lymphocytes. Other loci of concern include *ERAP1/2* and *IL23R*. The former encodes aminopeptidases that trim peptides for antigen presentation by MHC Class I protein whereas the latter codes for the interleukin-23 receptor (IL-23R). Activation of IL-23R triggers the differentiation of Th17 cells, which in turn secretes IL-17A, IL-17F and IL-22. Aside from NK cells and Th17 cells, immunocytes that are aberrantly activated in AS include dendritic cells, Th1 cells, Th2 cells, Th22 cells, cytotoxic T cells and B lymphocytes (11). These AS-related molecular and cellular abnormalities collectively lead to inflammation, erosion, and syndesmophyte formation at locations where ligaments, tendons and capsules are attached to the bone (12–15).

The therapeutic goals for early-stage AS are controlling inflammation (e.g. with non-steroidal anti-inflammatory drugs, anti-tumour necrosis factor (TNF) biologics, corticosteroids, sulfasalazine) and maintaining range of spinal movement (e.g. with exercise and physical therapy) (16–18). However, surgical intervention is inevitable for some patients with late-stage AS in order to improve pain and joint movement (19, 20). Despite the recent progress in drug development (e.g., the advent of anti-TNF biologics) for AS, the functional outcomes of many AS patients remain dissatisfying. In particular, the development of new therapy is challenged by the unclear pathogenesis of AS (21). Therefore, it is imperative to identify novel molecular targets for this potentially disabling condition.

Long non-coding RNAs (lncRNAs) are non-coding, regulatory RNAs longer than 200 nucleotides in length and have aroused intense interest from the medical and scientific community because of their regulatory functions in momentous biological functions (22–25). LncRNAs can modulate gene expression at the epigenetic (i.e., heritable changes in gene expression not caused through changes in DNA sequence by altering gene promoter DNA methylation and/or histone modification through recruiting DNA methyltransferase and/or chromatin-modifying enzymes to specific loci), transcriptional (e.g. by acting as a guide or decoy for specific transcription factors) and post-transcriptional [e.g. by sponging microRNAs (miRNAs)] level (26–30). Studies have shown that lncRNAs could act as critical regulators of diverse biological processes, such as cell proliferation, apoptosis, and the release of pro-inflammatory cytokines (22, 31–33). Moreover, similar to the key roles of miRNAs in human pathology (34–40), deregulated lncRNA expression has been documented in many kinds of diseases, such as degenerative diseases, tumors, cardiovascular disease, and autoimmune/inflammatory diseases (41–48). Recently, an increasing amount of evidence suggested that lncRNAs also actively take part in development of orthopedic diseases, including scoliosis, intervertebral disc herniation, arthritis and AS (49–51).

Emerging evidence support that dysregulation of non-coding RNAs, including miRNAs and lncRNAs, is pivotal to AS pathogenesis. In this connection, we previously reviewed the roles of miRNAs in AS, highlighting their involvements in modulating immune cell functions, such as cytokine response and T-cell survival (52). In the present review, we summarize the deregulation of another important class of non-coding RNA, namely lncRNAs, in AS in relation to their biological functions and molecular mechanisms, particularly sponging of miRNAs. We also discuss the potential utilization of serum and tissue lncRNAs as diagnostic or prognostic markers as well as druggable targets for AS.

## LncRNA Expression Profiling Studies in AS

In AS, differentially expressed lncRNAs have been identified at transcriptome-wide level by RNA sequencing or microarray in hip joint ligament, peripheral blood, peripheral blood mononuclear cells (PBMCs), or osteogenically differentiated mesenchymal stem cells (MSCs). Dysregulation of some lncRNAs have also been substantiated by reverse transcription-quantitative PCR (RT-qPCR).

### Hip Ligament

AS is associated with the abnormal ossification of the hip ligament. Zhang et al. performed microarray profiling to depict the mRNA, miRNA, and lncRNA expression landscapes in the hip joint ligament samples from AS patients compared to those from the femoral neck fracture patients as control (53). A total 574 mRNAs, 22 miRNAs and 661 lncRNAs were found to be deregulated in the AS group. Furthermore, the overexpression of CSNK1D-AS8 and NDRG1-AS6 and the downregulation of NR_045553, CD46-AS9 and SMYD5-AS2 in the AS group were confirmed by RT-qPCR.

### Peripheral Blood and PMBCs

Augmented inflammatory responsiveness and the associated pro-inflammatory cytokine release have been documented in PBMCs of AS patients (54, 55). Xu et al. performed RNA sequencing to identify AS-associated lncRNAs with blood samples from 3 AS patients and 3 normal controls (56). The authors identified 372 differently expressed lncRNAs (142 downregulated and 230 upregulated) in AS. RP11−837J7.4 and NALT1 were the top downregulated and upregulated differentially expressed lncRNAs in AS. Zhang et al. also used lncRNA microarray to profile lncRNA expression in PBMCs from 5 AS patients and 5 healthy controls (57). A total of 154 lncRNAs (83 downregulated and 71 upregulated) were found to be deregulated in AS. The significant upregulation of LOC101929023 and lncRNA H19 in AS was further confirmed. Similarly, Huang et al. conducted RNA sequencing to profile lncRNA expression in PBMCs from 15 AS patients and 15 age- and sex-matched healthy controls (58). A total of 270 lncRNAs (70 downregulated and 200 upregulated) were found to be differentially expressed in AS. The authors further confirmed the upregulation of ENST00000444046, NONHSAT183847.1 and NONHSAT118801.2 and downregulation of NONHSAT051856.2, NONHSAT205110.1 and NONHSAT105444.2 in AS samples. Importantly, they found that NONHSAT051856.2, NONHSAT205110.1, NONHSAT205110.1, NONHSAT183847.1, ENST00000444046, and NONHSAT118801.2 were correlated with clinical features of AS, in which the level of NONHSAT183847.1 and NONHSAT118801.2 were positively associated with disease severity.

### Osteogenically Differentiated MSCs

MSCs from AS patients have been shown to have a greater capacity for osteogenic differentiation. Xie et al. used microarray to profile mRNA and lncRNA expression in osteogenically differentiated MSCs from AS patients and healthy controls (59). There were 665 mRNAs and 520 lncRNAs found to be deregulated in AS. RT-qPCR was carried out to confirm that the downregulation of lnc-DTHD1-8, lnc-THBS2-3, lnc-GLRX5-2, lnc-FAM182B, lnc-NDUFS5, and lnc-MPDZ and the upregulation of lncMFN1-1, lnc-BLID-2, lnc-AYM1A-3, lnc-PIK3C2G-2, lnc-NOL6-4, and lnc-KLF14-1. Reconstruction of the coding-non-coding gene co-expression networks demonstrated that lnc-USP50-2, lnc-FRG2C-3, lnc-ZNF354A-1 and lnc-LIN54-1 were likely to be functionally involved in the AS-associated abnormal osteogenic differentiation.

The above-mentioned profiling studies collectively suggested that lncRNAs are pervasively deregulated in AS. It is expected that transcriptome-wide profiling in specific immune cell types pertinent to AS pathogenesis, such as T cells and peripheral blood-derived macrophages, will depict a more comprehensive picture of lncRNA deregulation in AS (**Tables 1**, **2**).

**Table 1:** LncRNAs expression profiles in ankylosing spondylitis.

Num	Method	Sample	Upregulated	Downregulated	Reference
1	Microarray RT-PCR	hip joint ligament	196 lncRNAs	465 lncRNAs	(52)
2	Microarra RT-PCR	PBMCs	71 lncRNAs	83 lncRNAs	(53)
3	Microarray RT-PCR	MSC	184 lncRNAs	336 lncRNAs	(55)
4	RNA sequencing RT-PCR	blood	230 lncRNAs	142 lncRNAs	(56)
5	RNA sequencing RT-PCR	PBMC	200 lncRNAs	70 lncRNAs	(54)

PBMCs, peripheral blood mononuclear cells.

**Table 2:** LncRNAs expression which was confirmed by qRT-PCR in ankylosing spondylitis.

Num	Method	Sample	Upregulated	Downregulated	Reference
1	Microarray RT-PCR	hip joint ligament	CSNK1D-AS8	NR_045553	(52)
			NDRG1-AS6	CD46-AS9	
				SMYD5-AS2	
2	Microarray RT-PCR	PBMCs	LOC101929023 H19		(53)
3	Microarray RT-PCR	MSC	lncMFN1-1	lnc-DTHD1-8	(55)
			lnc-BLID-2	lnc-THBS2-3	
			AYM1A-3	lnc-GLRX5-2	
			lnc-PIK3C2G-2	lnc-FAM182B	
			lnc-NOL6-4	lnc-NDUFS5	
			lnc-KLF14-1	lnc-MPDZ	
4	RNA sequencing RT-PCR	blood	NALT1	RP11−837J7.4	(56)
5	RNA sequencing RT-PCR	PBMC	ENST00000444046	NONHSAT051856.2	(54)
			NONHSAT183847.1	NONHSAT205110.1	
			NONHSAT118801.2	NONHSAT105444.2	

PBMCs, peripheral blood mononuclear cells.

### Mechanisms of Action of Functionally Important lncRNAs in AS

#### LncRNA-AK001085

Li et al. measured the expression of lncRNA-AK001085 in the serum of 117 AS patients and 76 healthy controls using RT-qPCR (60). They found that lncRNA-AK001085 was significantly downregulated in AS patients compared to healthy controls. Moreover, they demonstrated that exercise level, occupational activity and cigarette smoking were associated with lncRNA-AK001085 level. LncRNA-AK001085 level was also negatively correlated with erythrocyte sedimentation rate (ESR), C-reactive protein (CRP), Ankylosing Spondylitis Disease Activity Score (ASDAS), and Bath Ankylosing Spondylitis Disease Activity Index (BASDI). Receiver operating characteristic (ROC) curve analysis showed an area under the curve (AUC) of 0.868 with a specificity of 93.6% and a sensitivity of 62.9% for serum lncRNA-AK001085 in discriminating cases from controls. These data suggested that lncRNA-AK001085 might be a promising diagnostic marker for AS.

#### NKILA

Gai et al. demonstrated that plasma level of NKILA (NF-κB Interacting LncRNA) was higher in the active AS group as compared with the inactive AS group and the control group (61). Cases with high NKILA level also required longer therapy course. The expression of transforming growth factor (TGF)-β (a cytokine with increased expression in AS) was also significantly positively correlated with NKILA expression in the active AS group. In this respect, TGF-β has been shown to induce Th17 cell differentiation in the presence of IL-6 (62). These results suggested that NKILA may be functionally related to TGF-β signaling and may serve as a prognostic marker in AS.

#### H19

Zhang et al. showed that the lncRNA H19 was significantly upregulated in AS cases as compared with healthy controls (57). Knockdown of H19 suppressed vitamin D receptor (VDR) and miR-675-5p expression and promoted miR-22-5p expression in PBMCs. Moreover, knockdown of H19 inhibited the mRNA expression of interleukin (IL)-23 and IL-17A (a T helper 17 effector cytokines) but not TNF-α. In line with this, gain-of-function experiments demonstrated that overexpression of H19 promoted IL-17 and VDR expression and inhibited miR-22-5p expression. Functionally, H19 regulated inflammation by acting as a competing endogenous RNA along the VDR-IL-23/17A signaling axis *via* interactions with miR-675-5p and miR-22-5p. These data suggested that H19 may be a therapeutic target for AS.

#### TUG1

Lan et al. demonstrated that level of the lncRNA TUG1 (Taurine Upregulated Gene 1) was decreased in AS patients as compared with healthy controls in sacroiliac biopsies and serum (63). The AUC for biopsy and serum TUG1 level in discriminating cases from controls were 0.8911 and 0.7968, respectively. Importantly, TUG1 level was negatively correlated with CRP and disease activity in AS patients. Moreover, TUG1 level was associated with smoking habit and patients’ course. The authors’ data suggested that TUG1 expression was downregulated in AS and its low expression was associated with higher disease activity, more frequent rehospitalization, and longer treatment course. These data suggested that TUG1 may serve as a diagnostic as well as a prognostic marker for AS.

#### MEG3

Liu et al. showed that the lncRNA MEG3 (Maternally Expressed Gene 3) was downregulated in sacroiliac joint biopsies and serum of AS patients compared to the healthy controls (64). The AUC for biopsy and serum MEG3 in discriminating cases from controls were 0.8862 ad 0.7484, respectively. MEG3 level was closely associated with the disease activity, in which AS patients with lower MEG3 level were hospitalized for a longer duration and re-hospitalized more frequently. Consistently, another study reported that MEG3 was downregulated in the serum of AS patients as compared with controls (65). The levels of TNF-α, IL-6, and IL-1β were also increased in the AS patients, in which MEG3 level was negatively correlated with these pro-inflammatory cytokines. Functionally, restored expression of MEG3 suppressed TNF-α, IL-6, and IL-1β expression whereas knockdown of MEG3 produced the opposite effects in fibroblast-like synovial cells. Mechanistically, MEG3 was found to sponge miR−146a, which was upregulated in AS patients and was able to induce TNF-α, IL-6, and IL-1β expression. These findings indicated that aberrant downregulation of MEG3 could promote inflammatory response through increasing the abundance of miR−146a. Similarly, Ma et al. showed that enforced expression of MEG3 led to the sponging of let-7i, resulting in the restoration of sclerostin expression and inhibition of pro-inflammatory cytokine release in AS fibroblasts (66). These studies collectively suggested that MEG3 downregulation may serve as both a diagnostic and prognostic marker for AS. Restoring MEG3 expression could also be a novel therapeutic approach to curb AS progression.

#### LINC00311

Zhong et al. demonstrated that plasma LINC00311 level was higher in AS cases as compared with healthy controls (67). In this connection, plasma LINC00311 could distinguish cases from controls with an AUC of 0.9041 and was positively associated with ESR, CRP, BASDAI and ASDAS. The plasma level of LINC00311 in AS patients was also decreased after treatment. Furthermore, cases with high plasma LINC00311 level had a higher rate of rehospitalization. These results suggested that LINC00311 was upregulated in AS and could predict disease activity and recurrence.

#### LOC645166

Yu et al. demonstrated that LOC645166 was one of the most downregulated lncRNAs in T cells from AS patients as compared to those from healthy controls (68). Enforced expression of LOC645166 suppressed the expression of IL-23p19 (a subunit of IL-23) and inhibited JAK2/STAT3 signaling induced by phorbol 12-myristate 13-acetate or anti-CD28/CD3 antibodies in Jurkat cells (an immortalized line of human T lymphocytes). Mechanistically, they found that LOC645166 could bind with K63-linked polyubiquitin chains and inhibited their recruitment to the IKK complex to diminish phosphorylation of IKK2, resulting in NF-κB inhibition. Knockdown of LOC645166 induced NF-kB activation produced the opposite effects. These data suggested that LOC645166 played important roles in development of AS through regulating NF-κB in T cells.

#### Lnc-ITSN1-2

Li and Zhou demonstrated that Lnc-ITSN1-2 (long noncoding RNA intersectin 1-2) level in PBMCs was upregulated in AS patients and positively correlated with CPR and IL-1β, ASDAS and BASDAI score (69). Furthermore, the expression of lnc-ITSN1-2 was found to be decreased in AS patients treated with adalimumab (an anti-TNF biologic), in which responders showed a drastic decline. These data suggested that PBMC lnc-ITSN1-2 might be used as a surrogate marker of responsiveness to anti-TNF biologics in AS patients.

## Conclusions and Future Perspectives

**Figure 1:**
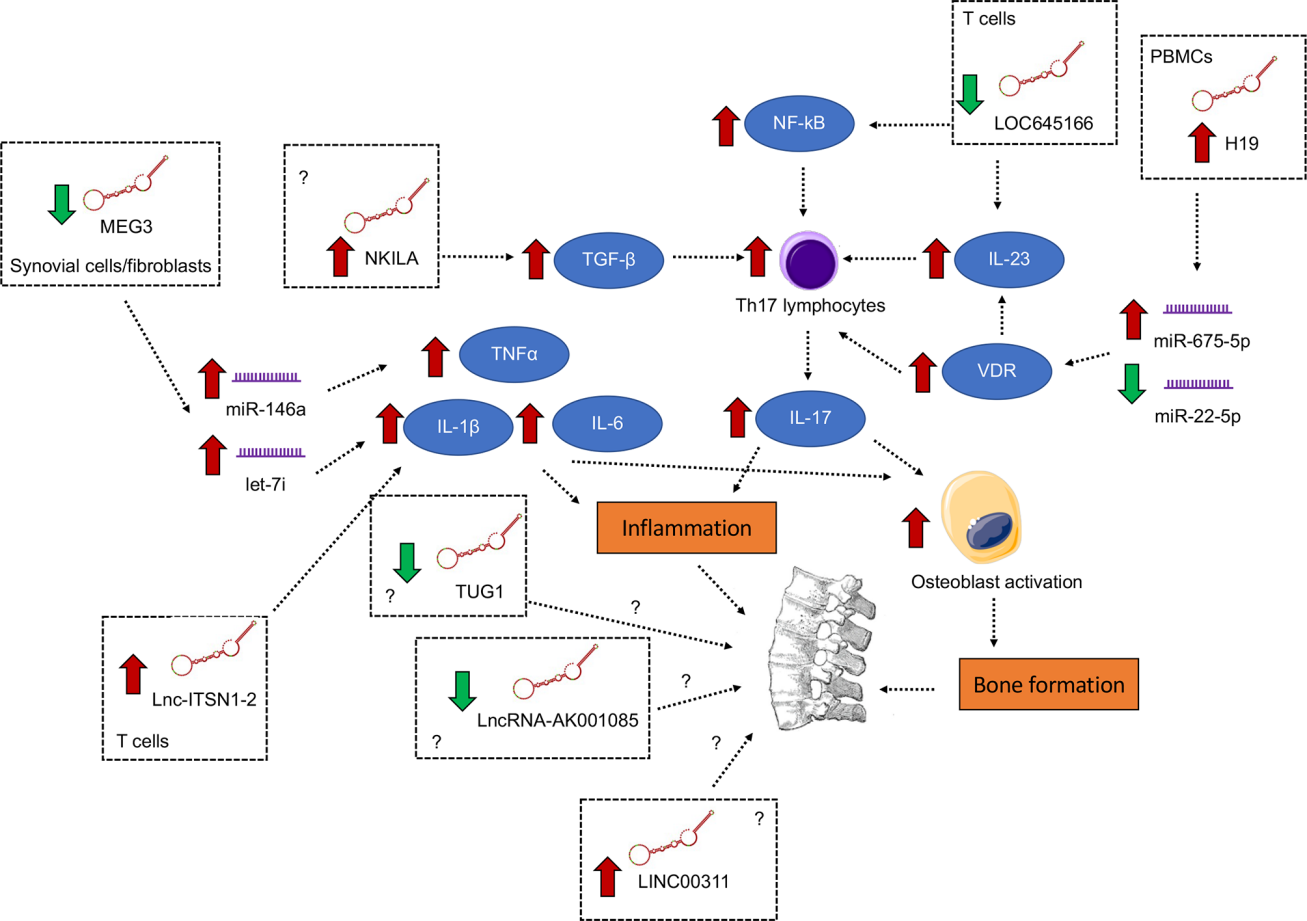
Differentially expressed lncRNAs with functional importance in the pathogenesis of ankylosing spondylitis. The signaling converge on the induction of pro-inflammatory cytokines IL-1β, TNF-α and IL-6 and the augmentation of the IL-23/IL-17 axis, both of which contribute to inflammation and abnormal bone formation.

AS is an autoimmune disease with poorly-defined etiology and pathogenesis (70–72). Thus, more effort is needed to elucidate the underlying pathobiology so as to develop novel, mechanism-driven therapeutics. LncRNAs function as an important class of regulatory element of the genome. In AS, lncRNAs were found to be pervasively deregulated in different tissues or cell types, including hip ligament, whole blood, PBMCs, T cells and osteogenically differentiated MSCs, and participated in the regulation of inflammatory signaling, particularly the induction of pro-inflammatory cytokines IL-1β, TNF-α and IL-6 and the augmentation of the IL-23/IL-17 axis (**Figure 1**). In this connection, aside from perpetuating the inflammatory condition, IL-17 from T cells and IL-1 and IL-1β, TNF-α and IL-6 from monocytes have been shown to stimulate osteoblasts to induce abnormal bone formation (73), a characteristic of AS. It is hopeful that, with more mechanistic studies, more functionally important lncRNAs and their downstream pathways which are key to AS pathogenesis will be identified.

As potential biomarkers for AS diagnosis, multiple circulating lncRNAs (increased levels of NKILA, H19, LINC00311 and Lnc-ITSN1-2; reduced levels of lncRNA-AK001085, TUG1 and MEG3) were reported to possess the ability to distinguish the cases from controls with decent sensitivity and specificity. Many of these circulating lncRNAs also correlate well with disease severity and could predict rehospitalization or response to treatment and are therefore of prognostic value. Nevertheless, similar to other biomarkers, these lncRNA markers should be validated in larger cohorts and in different populations. Moreover, the methods for lncRNA quantification need to be standardized with the lncRNA normalized using a proper internal control for maximising the reproducibility across different laboratories. Last but not least, combining these lncRNAs with genetic testing as well as panels of existing and emerging markers is expected to further improve the sensitivity and specificity.

Therapeutically, targeting upregulated lncRNAs by antisense RNA-, RNA interference-, or CRISPR (clustered regularly interspaced short palindromic repeat)-based therapeutics might offer new methods to control AS progression. However, delivering these therapies in a tissue- or cell type-specific manner is required for avoiding off-target effects, yet remains challenging. Resolving these outstanding issues is needed before the clinical use of lncRNAs as biomarkers or therapeutic targets can be fully embraced.

## Author Contributions

RS, XWa, XS, BZ, XZ, and XWo drafted and wrote the manuscript. XG, SW, MC, and WW revised the manuscript. XWa participated in the design of the review. All authors read and approved the final manuscript.

## Conflict of Interest

The authors declare that the research was conducted in the absence of any commercial or financial relationships that could be construed as a potential conflict of interest.

## Publisher’s Note

All claims expressed in this article are solely those of the authors and do not necessarily represent those of their affiliated organizations, or those of the publisher, the editors and the reviewers. Any product that may be evaluated in this article, or claim that may be made by its manufacturer, is not guaranteed or endorsed by the publisher.
